# Feasibility of using mixtures of silicone elastomers and silicone oils to model the mechanical behaviour of biological tissues

**DOI:** 10.1177/0954411914540138

**Published:** 2014-07

**Authors:** S Mohammad Hassan Ahmadzadeh, David WL Hukins

**Affiliations:** 1 School of Mechanical Engineering, University of Birmingham, Birmingham, UK; 2 Department of Biomedical Engineering, Imperial College London, London, UK

**Keywords:** Modelling biological tissues, silicones, Young’s modulus

## Abstract

Mixtures of silicone elastomer and silicone oil were prepared and the values of their Young’s moduli, *E*, determined in compression. The mixtures had volume fractions, ϕ, of silicone oil in the range of 0–0.73. Measurements were made, under displacement control, for strain rates, $\overset{\cdot }{\varepsilon }$, in the range of 0.04–3.85 s^−1^. The behaviour of E as a function of ϕ and $\overset{\cdot }{\varepsilon }$ was investigated using a response surface model. The effects of the two variables were independent for the silicones used in this investigation. As a result, the dependence of *E* values (measured in MPa) on ϕ and $\overset{\cdot }{\varepsilon }$ (s^−1^) could be represented by $E=0.57-0.75\phi +0.01\;\log_{e}(\overset{\cdot }{\varepsilon })$. This means that these silicones can be mixed to give materials with *E* values in the range of about 0.02–0.57 MPa, which includes *E* values for many biological tissues. Thus, the mixtures can be used for making models for training health-care professionals and may be useful in some research applications as model tissues that do not exhibit biological variability.

## Introduction

This article reports an investigation into the feasibility of blending silicone elastomers and silicone oils to produce materials that can be used to make physical models of biological tissues; such models are becoming increasingly important for training health-care professionals.^1–3^ For example, they can replace human cadavers in medical education and be used to practice procedures (such as injection or surgery) before they are performed on living patients. The purpose of this article is very different from the development of materials for patient care; its aim is solely to aid making models for training purposes that are not intended for implantation. Synthetic materials may also be useful in research to mimic the mechanical behaviour of natural tissues without the biological variability.^3–5^ Also the purpose of this article is not to investigate the interactions of silicone oils and silicone elastomers or to investigate the mechanisms by which the oils can modify the properties of the elastomer. Its purpose is simply to develop empirical rules that enable materials to be formulated whose mechanical properties resemble those of natural tissues.

In this article, the ability of a material to deform when subjected to an applied force was considered to be the most important factor in producing a model material that resembles real biological tissue. For some research applications, it may be necessary to consider other properties such as yield strength and energy absorbed to yield.^4^ Therefore, a series of mixtures of a silicone oil and a silicone elastomer have been produced with different compositions and their Young’s modulus determined, as a measure of the deformability of the material. Since silicones are viscoelastic, the Young’s modulus of each mixture was measured at different strain rates.^6^

## Materials and methods

### Materials

The experiments were performed on PlatSil^©^ Gel-10 silicone elastomer (Mouldlife Ltd, Suffolk, UK) blended with silicone oil (Smith’s Deadener from Mouldlife Ltd). According to the manufacturer’s data sheets for these products, mixtures of this kind are intended for modelling tissues for theatrical make-up. The elastomer was supplied as two parts (A and B) that needed to be mixed and cured to make the pure elastomer. The silicone oil was incorporated during the mixing stage to make the blended product.

Equal amounts of parts A and B were mixed together at room temperature (25 °C), according to the supplier’s instructions. Silicone oil was added to the mixtures of parts A and B in order to make specimens with different silicone oil concentrations. All compositions were controlled by weighing with a precision of 0.1 mg. Components were mixed together by hand for about 5 min at room temperature (25 °C) in polypropylene beakers (capacity 250 mL). It was found that rapid mixing reduced the number of air bubbles formed; further air bubbles were removed during the specimen preparation stage. Eight different mixtures of silicone elastomer and silicone oil were prepared with the compositions listed in Table 1.

**Table 1.** Composition of mixtures of silicone elastomer and silicone oil.

Mass fraction of silicone oil (ψ)	PlatSil Gel-10 A (kg)	PlatSil Gel-10 B (kg)	Silicone oil (kg)	Volume fraction of silicone oil (ϕ)
0	0.1	0.1	0	0
0.1	0.09	0.09	0.02	0.11
0.2	0.08	0.08	0.04	0.22
0.3	0.07	0.07	0.06	0.33
0.4	0.06	0.06	0.08	0.43
0.5	0.05	0.05	0.1	0.53
0.6	0.04	0.04	0.12	0.63
0.7	0.03	0.03	0.14	0.73

The volume fraction of silicone oil, ϕ, was calculated for each mixture from

(1) $$\phi =\frac{\rho_{\mathrm{se}}\psi }{\left\{\rho_{\mathrm{so}}(1-\psi )+\rho_{\mathrm{se}}\psi \right\}}$$

where $\rho_{\mathrm{se}}$ is the density of silicone elastomer (an equal mixture of parts A and B, 1100 kg m^−3^, according to the manufacturer’s data sheet); $\rho_{\mathrm{so}}$ and ψ are the density (967 kg m^−3^) and mass fraction of silicone oil, respectively (listed in Table 1). Equation (1) has been published previously,^7^ has been used many times previously and arises directly from the definition of volume fraction.^8^ Calculated values of ψ are listed in Table 1.

### Specimens

The dimensions of the cylindrical specimens for compression testing complied with British standards for testing rubbers.^9^ They are identical to those used previously for compression testing of silicone elastomers, and the methods used to make them are based on those reported previously.^6^

Specimens (diameter: 29 mm, thickness: 13 mm) were pressed into a Teflon mould. For each composition of the mixture, 10 cylindrical specimens were pressed into the mould under 500 N load at room temperature (25 °C) using a Lloyd Instruments machine (Model L6000R with a 1-kN load cell; Lloyd Instruments Ltd, Fareham, UK). The load was applied in three stages, each lasting 15 min, in order to remove air bubbles. At the end of each stage, the force was removed from the mould for 5 min and the next load was applied after that 5 min. In previous studies of silicone elastomers, where air bubble formation was not a problem, a single application of a 50-N load was used.

### Mechanical testing

Compression tests were performed on the specimens listed in Table 1 (10 samples for each composition) using a material testing machine (ELF 3200; Bose Corporation, ElectroForce Systems Group, Eden Prairie, MN, USA) with a load cell of 225 N (nominal precision ± 0.005 N) and a displacement transducer with full scale 6.5 mm. All tests were controlled by WinTest software (Bose Corporation, ElectroForce Systems Group).

Uniaxial compression was applied under displacement control. Each specimen was compressed to 5 mm (corresponding to a strain value of about 0.4) through a brass plate, the diameter of which was slightly greater (30 mm) than the diameter of cylindrical specimens (29 mm). The specimens were mounted on the test rig that was attached to the base of testing machine, and the surface of the cylindrical specimen was placed in contact with the circular plate on the actuator and aligned with it, as recommended by other studies,^10^ to apply a uniform stress and to reduce non-uniform stress at the specimen edges.^11^ The test was started from zero load and displacement and each specimen was compressed under displacement control at rates of 0.5, 10, 30 and 50 mm/s, which are equivalent to strain rates of 0.04, 0.77, 2.31 and 3.85 s^−1^, respectively. Different strain rates were applied in order to investigate the effect of strain rate on the mechanical properties of the material. In each compression test, once the specimen was compressed to 5 mm (a strain value of about 0.4), it was then unloaded and returned to its initial position at the corresponding strain rate.

The measured load was converted to engineering stress and the displacement to engineering strain. The origin of the load–displacement (and, therefore, of the stress–strain) curve was defined as the point at which the testing machine started to register an increase in load as the actuator approached the specimen. A second-order polynomial was fitted to the stress–strain curve and the Young’s modulus defined to be the slope of this curve at a strain value of 0.2, since all curves had a linear portion centred around this region.

### Statistical analysis

The behaviour of E (the response) as a function of ϕ and strain rate, $\overset{\cdot }{\varepsilon }$, (the variables) (i.e. how E depends on ϕ and $\overset{\cdot }{\varepsilon }$) was modelled using response surface methodology.^12–14^ A surface model defined by

(2) $$\begin{array}{cc} E= & c_{1}+c_{2}\phi +c_{3}\;\log_{e}(\overset{\cdot }{\varepsilon })+c_{4}\phi^{2}\\ +c_{5}{\text{log}}_{e}^{2}(\overset{\cdot }{\varepsilon })+c_{6}\phi \log_{e}(\overset{\cdot }{\varepsilon }) \end{array}$$

was fitted to the results; *c*_1_–*c*_6_ are the coefficients that gave the best fit to the data points. The coefficients of the model, the standard errors (SEs) of the slopes of the regression lines and the coefficient of determination (*R*^2^) that measure the correlation between the response and the variables were calculated by the method of least-squares.^12,15^

The significance of the terms in the model was assessed by analysis of variance^12–15^ and testing the null hypothesis that the coefficients were 0. These hypotheses were rejected (i.e. a significant fit was obtained) if the probability that they were false, *p*, was less than 0.05. The residuals for the model (i.e. the difference between observed and fitted values of *E*) were determined to examine the correlation between the errors, constant variance of errors, normality of the errors, missing of higher order terms in the model and the presence of outliers.^16^ All statistical analysis and calculations were performed using a spreadsheet (Excel 2007; Microsoft, Reading, UK) and Minitab software (Minitab^®^ version 16 Statistical Software; Minitab Ltd, Coventry, UK).

## Results

**Figure 1.**
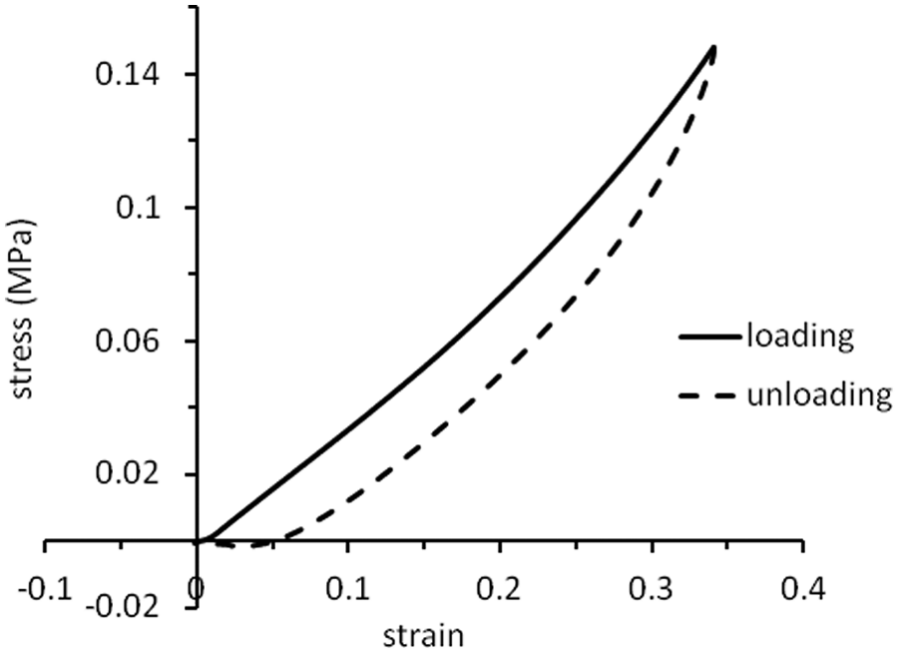
Stress–strain curve for a mixture of silicone elastomer and silicone oil (volume fraction of silicone oil, ϕ=0.11); the upper (continuous) curve represents loading and the lower (dashed) curve represents unloading. Stress is measured in megapascal.

Figure 1 shows a typical stress–strain curve for a sample of silicone elastomer mixed with silicone oil. The upper curve represents the loading phase of the test and the lower curve shows the unloading phase, that is, the samples exhibited hysteresis, demonstrating their viscoelastic properties. All measurements were made on the upper (loading) curve. In all cases, this curve is slightly concave but the central region (around a strain of about 0.2) is close to being linear. It was, therefore, considered reasonable to represent the response of the samples by a single value of the Young’s modulus, *E*, measured at a strain ε=0.2.

After fitting equation (2) to the experimental results, it was found that the dependence of *E* values (MPa) on volume fraction of silicone oil, ϕ, and strain rate, $\overset{\cdot }{\varepsilon }$ (s^−1^), could be represented by

(3) $$E=0.57-0.75\phi +0.01\;\log_{e}(\overset{\cdot }{\varepsilon })$$

**Figure 2.**
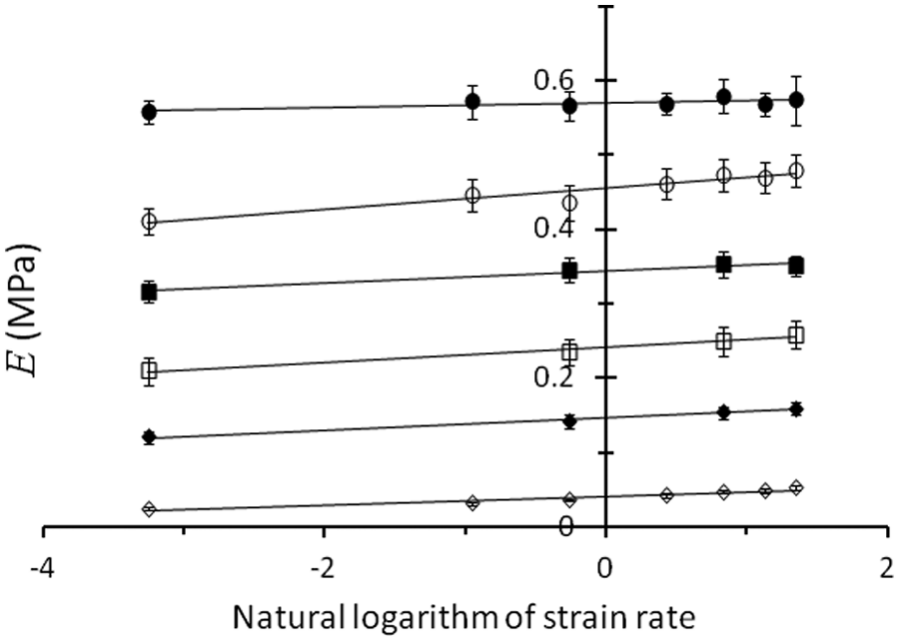
Young’s modulus, *E* (MPa), plotted against the natural logarithm of the strain rate, $\text{lo}{\text{g}}_{e}(\overset{\cdot }{\varepsilon })$, when $\overset{\cdot }{\varepsilon }$ is measured in s^−1^, for mixtures with a range of values for the volume fraction, ϕ, of silicone oil: 0 (i.e. pure elastomer), filled circles; 0.11, open circles; 0.33, filled squares; 0.43, open squares; 0.53, filled diamonds; 0.73, open diamonds. Mean values of *E* are plotted; error bars represent standard deviations.

**Figure 3.**
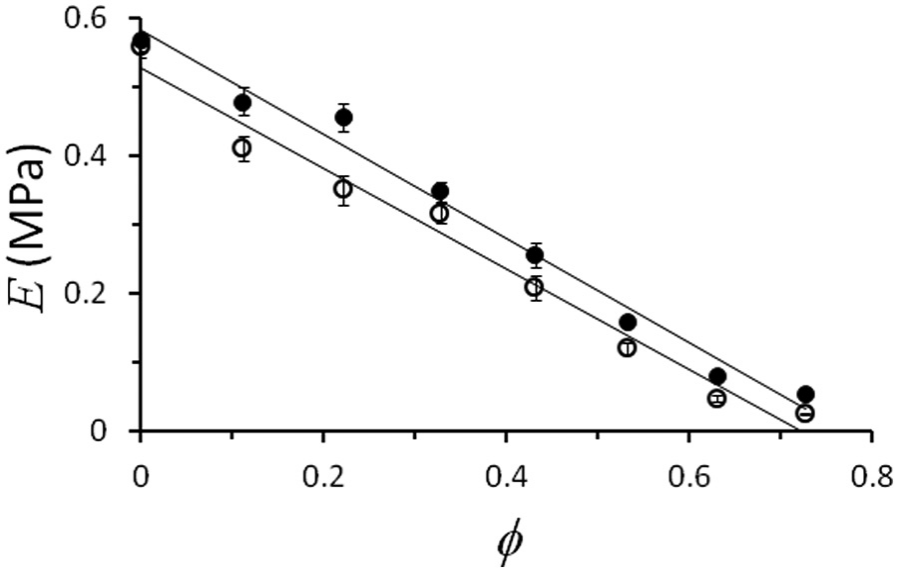
Young’s modulus, *E* (MPa), plotted against the volume fraction, ϕ, of silicone oil for fast (3.85 s^−1^; filled circles) and slow (0.04 s^−1^; open circles) values of the strain rate, $\overset{\cdot }{\varepsilon }$. Error bars represent standard deviations; where error bars are not shown, they are smaller than the data points. Regression lines plotted through each set of data points have *R*^2^ = 0.98.

Analysis of variance on the coefficients of the surface model showed that non-linear (coefficients $c_{4}$ and $c_{5}$) and cross (coefficient $c_{6}$) terms were insignificant. This means that the dependence of *E* on ϕ and $\overset{\cdot }{\varepsilon }$ are uncorrelated (no cross term in the equation) and linear. Figure 2 shows the dependence of *E* on $\text{lo}{\text{g}}_{e}(\overset{\cdot }{\varepsilon })$ for a range of sample compositions. Figure 3 shows the dependence of *E* on ϕ for the fastest (3.85 s^−1^) and slowest (0.04 s^−1^) values of $\overset{\cdot }{\varepsilon }$.

## Discussion

Equation (3) enables the silicone oil and silicone elastomer used here to be blended to give materials with a Young’s modulus in the range of about 0.02–0.57 MPa. Many biological tissues have a Young’s modulus within this range and so can be modelled using the materials used here in the correct proportions. Examples include brain (0.07),^17^ bladder (0.25),^18^ breast (0.03)^19^ and prostate (0.06).^19^ In practice, Young’s modulus values measured for silicones can be slightly different in tension and compression; however, the difference is much less than the variability in typical measurements of Young’s modulus from biological tissues.^20^

The stress–strain curve shown in Figure 1 is non-linear, as usually observed for silicones and other elastomers.^21^ Extension occurs by alignment of their polymer molecules, so energy is expended to decrease the entropy of the material; relaxation is accompanied by an increase in entropy and so is largely driven by entropy rather than energy changes.^22^ There is no reason to suppose that stress–strain curves for such materials need to be linear.

The hysteresis apparent in Figure 1 shows that some of the mechanical energy imparted to the mixtures in compression was lost, rather than being stored for subsequent recoil, that is, the mixtures of silicone elastomer and silicone oil were viscoelastic. Many biological tissues are also viscoelastic and so, strictly speaking, their Young’s modulus is a complex number in which the real part represents the elastic (storage) modulus and the imaginary part represents the viscous (loss modulus).^23^ However, the loss modulus has not been routinely measured for many biological tissues. Furthermore, the purpose of this article is to guide the production of materials whose handling properties correspond reasonably well to those of natural tissues. For these reasons, measurement of Young’s modulus (strictly speaking, the magnitude of the complex modulus) was considered to be an adequate guide. In the future, the production of more sophisticated tissue models may require the measurements of the real and imaginary parts as, for example, has been performed for implantable grades of silicones and other elastomers.^6,20,24,25^

Silicone oil may be used as a plasticizer to modify the properties of silicone elastomer.^26^ It presumably acts as a plasticizer by separating the polymer chains of the elastomer phase, so that less energy is required to reorganize them, and thus making the material more compliant. This is the mechanism whereby liquids incorporated into polymeric materials usually make them more compliant.^27^

If the function of the silicone oil was simply to dilute the proportion of silicone elastomer in the mixtures, they might be expected^23^ to obey a ‘law of mixtures’ so that *E* would be given by

(4) $$E=\phi E_{\mathrm{so}}+(1-\phi )E_{\mathrm{se}}\approx E_{\mathrm{se}}(1-\phi )$$

In equation (4), $E_{\mathrm{so}}$ and $E_{\mathrm{se}}$ are the Young’s moduli of the silicone oil and the silicone elastomer, respectively; the approximate equality arises because $E_{\mathrm{so}}<<E_{\mathrm{se}}$ since silicone oil is a liquid. If equation (4) adequately represents the behaviour of these mixtures, it predicts that the results of Figure 1 should show a linear dependence on ϕ with a slope of $-E_{\mathrm{se}}$. The regression lines of Figure 3 show a close fit to the experimental data with a negative slope. However, the slopes of the curves for fast (−0.76 MPa) and slow loading (−0.73 MPa) are substantially different to $-E_{\mathrm{se}}$ (−0.57 and −0.56 MPa, respectively). Therefore, a simple law of mixtures cannot completely specify the behaviour of these mixtures of silicone oil and silicone elastomer.

## Conclusion

Silicone oils and silicone elastomers can be blended to give mixtures whose Young’s modulus is suitable for building physical models of biological tissues. When a particular silicone oil (Smith’s Deadener from Mouldlife Ltd) is mixed with a particular silicone elastomer (PlatSil Gel-10, Mouldlife Ltd) at a volume fraction, the Young’s modulus of the mixture (MPa) is given by $E=0.57-0.75\phi +0.01\;\log_{e}(\overset{\cdot }{\varepsilon })$, where $\overset{\cdot }{\varepsilon }$ represents strain rate (s^−1^).
